# Antimicrobial Susceptibility of *Escherichia coli* Isolates Causing Community-Acquired Urinary Tract Infections: Comparison of Methods

**DOI:** 10.3390/microorganisms13020231

**Published:** 2025-01-22

**Authors:** Alexander Machado Cardoso, Vinicius Ribeiro Flores, Gabriel Gomes do Rosario, Juliana Barbosa Succar, Lidiane Coelho Berbert, Maria Clara de Freitas Oliveira, Anna Luiza Bauer Canellas, Marinella Silva Laport, Cláudia Rezende Vieira Mendonça Souza, Thiago Pavoni Gomes Chagas, Rubens Clayton da Silva Dias, Fabio da Silva de Azevedo Fortes, Flávia Lúcia Piffano Costa Pellegrino

**Affiliations:** 1 Laboratory of Environmental Biotechnology, Faculty of Biological and Health Sciences (FCBS), Rio de Janeiro State University (UERJ), Rio de Janeiro 23070-200, Brazil; julisuccar@hotmail.com (J.B.S.); lidy_berbert@hotmail.com (L.C.B.); 2 National Institute of Technology (INT), Rio de Janeiro 20081-312, Brazil; viniciusflores42@gmail.com; 3 Integrated Laboratories for Research on Antimicrobial-Resistant Bacteria and Galenic Development (LIPE), Faculty of Biological and Health Sciences (FCBS), Rio de Janeiro State University (UERJ), Rio de Janeiro 23070-200, Brazil; gabrielgomes8518@gmail.com (G.G.d.R.); mariaclara.mcf68@gmail.com (M.C.d.F.O.); 4 Laboratory of Molecular and Marine Bacteriology, Paulo de Góes Institute of Microbiology, Federal University of Rio de Janeiro (UFRJ), Rio de Janeiro 21941-902, Brazil; annaluiza@micro.ufrj.br (A.L.B.C.); marinella@micro.ufrj.br (M.S.L.); 5 Department of Pathology, Fluminense Federal University (UFF), Niterói 24220-008, Brazil; claudia_souza@id.uff.br (C.R.V.M.S.); tpgchagas@id.uff.br (T.P.G.C.); 6 Biomedical Institute, Federal University of the State of Rio de Janeiro (UNIRIO), Rio de Janeiro 20211-040, Brazil; rubens.dias@unirio.br; 7 Laboratory of Cellular and Molecular Therapy and Physiology (LTFCM), Faculty of Biological and Health Sciences (FCBS), Rio de Janeiro State University (UERJ), Rio de Janeiro 23070-200, Brazil; fabio.fortes.uerj@gmail.com

**Keywords:** antimicrobial susceptibility determination, disk-diffusion test, CLSI, BrCAST, automated systems, uropathogenic *Escherichia coli*, urinary tract infection

## Abstract

Due to bacterial resistance to antimicrobials, antibiotic therapy for urinary tract infections (UTIs) has become a major challenge for clinicians. The present work aimed to compare the antimicrobial susceptibility profiles of 53 uropathogenic *Escherichia coli* (UPEC) isolates, assessed using the disk diffusion method and two automated systems (PHOENIX BD™ and VITEK2), with interpretations based on CLSI and BrCAST guidelines. Twenty-five antibiotics were tested to assess differences in susceptibility profiles. Statistical tools, including Kappa coefficient analysis and chi-square tests, were applied to assess concordance and significance between methods. Among the main discrepancies found, BrCAST has classified a greater number of UPEC isolates as resistant to more than half of the antibiotics tested by the disk diffusion method, when compared to CLSI. Although faster, the PHOENIX BD and VITEK2 automated systems exhibited significant discrepancies, with divergences observed for half of the antimicrobials tested. Both automated methods showed discrepancies compared to the disk diffusion method under CLSI and BrCAST guidelines. PHOENIX BD classified some isolates resistant by DD/CLSI as susceptible, while VITEK2 misclassified 25% to 50% of the antimicrobials tested. Conversely, VITEK2 also classified some isolates susceptible to DD/CLSI as resistant to 25% of the antimicrobials tested. Regarding DD/BrCAST, PHOENIX BD classified resistant isolates as susceptible (to 50% of the antimicrobials tested). In comparison, VITEK2 classified resistant isolates as susceptible and susceptible isolates as resistant (25% of the antimicrobials for both). These findings highlight the need for careful selection of susceptibility testing methods, as variations in interpretive criteria between CLSI and BrCAST could impact clinical decision-making. This study underscores the importance of methodological consistency in accurately informing antibiotic therapy in UTI management, especially in the face of rising resistance.

## 1. Introduction

Uropathogenic *Escherichia coli* (UPEC) is the etiological agent of about 80–90% of urinary tract infection (UTI) cases in the world [1]. Treatment includes the empirical use of antimicrobials, mostly indicated based on symptoms, but without carrying out previous urine culture and antimicrobial susceptibility tests [2]. In severe cases, particularly those originating in the community setting, susceptibility testing of uropathogens to antimicrobials is often not conducted. This greatly increases the chances of therapeutic failure, risk of antibiotic indiscriminate use, and selective pressure under resistant bacterial strains that can spread and propagate the phenomenon of resistance [3,4,5].

Tests to determine the susceptibility of specific bacterial pathogens to antimicrobials, whether manual or automated, are precious tools to guide therapy [6]. Guidelines for standardization and interpretation for antimicrobial susceptibility tests and cutoff points, such as those provided by the American (CLSI, Clinical and Laboratory Standards Institute), by the European (EUCAST, European Committee on Anti-microbial Susceptibility Testing), or by the Brazilian (BrCAST, Brazilian Committee for Antimicrobial Susceptibility Testing) committee, are useful as efficient parameters to guide the choice of antimicrobial agents used in the treatment of patients with bacterial infections including UTIs [7].

About a decade ago, Brazil did not have its own standardization committee to interpretate cut-off points obtained in susceptibility tests. For many years, CLSI manual was used for this by most clinical and microbiology research laboratories. After being recognized by EUCAST and approved by the Ministry of Health, in 2014, BrCAST started its activities in Brazil and can be used in all public and private microbiology laboratories in Brazil [4]. Since December 2019, the use of the BrCAST manual has been mandatory for all Brazilian clinical laboratories. The Brazilian Committee on Antimicrobial Susceptibility Testing, named BrCAST, is a committee designated and ratified by the Brazilian Society of Clinical Analysis, the Brazilian Society of Infectious Diseases, the Brazilian Society of Microbiology and the Brazilian Society of Clinical Pathology and Laboratory Medicine. BrCAST acts to standardize and improve the quality of sensitivity tests across the country and combat antimicrobial resistance. BrCAST document is based, with exceptions, in the cutoff points of version 11.0, 2021 of EUCAST the European manual. In this way, we can say that BrCAST would be a Brazilian version of EUCAST [4,7,8,9].

Clinical and Laboratory Standards Institute (CLSI), formerly known as the National Committee for Clinical Laboratory Standards (NCCLS), can still be considered the most used manual worldwide for interpretation of antimicrobial susceptibility testing [8]. Since 2005, CLSI was the single guideline used in Brazil until the implementation of BrCAST. Although CLSI had been used in the clinical and research laboratories in the large centers from Brazil for decades, in many parts of the country there was difficulty obtaining updated versions of the manual and with reading the English language. Thus, BrCAST guidelines were set as the new standard for AST in Brazil [4]. BrCAST is a manual in Portuguese available for free on its website [9]. Like EUCAST, BrCAST presents cut-off points based on epidemiological studies of bacterial infections and pharmacokinetic-pharmacodynamic properties of the antimicrobial drugs tested; Unlike CLSI, EUCAST has representation in several countries, including Brazil [4,8,9,10,11].

The present study shows a comparative analysis of different methods for determining the antimicrobial susceptibility of UPEC isolates (BD PHOENIX and VITEK2 automated systems and the Disk diffusion testing), using BrCAST (Version 13.0) and CLSI for interpretation. This topic is of critical importance, as bacterial resistance to antimicrobials is recognized by the World Health Organization (WHO, Geneva, Switzerland) as a major global public health challenge [1].

## 2. Materials and Methods

### 2.1. Bacterial Samples

Fifty-three bacterial isolates recovered from the urine of 53 distinct patients with urinary tract infections and assisted at a public hospital in the metropolitan region of Rio de Janeiro, Brazil, from May to September 2019, were analyzed in the present study. Informed consent was not required for this study because patient samples were anonymized, and no personal data were collected, as per the guidelines of the Ethics Committee (Research Ethics Committee No. 2.920.186/CAAE No. 95984018.6.0000.5243).

### 2.2. Bacterial Identification

Bacterial isolates were identified by Matrix-Assisted Laser Desorption/Ionization Time-of-Flight Mass Spectrometry (MALDI-TOF MS). Isolates were grown overnight on LB agar and the bacterial biomass was directly disposed onto an MSP 96 target polished steel plate (Bruker Daltonics, Bremen, Germany) and overlaid with 1 µL of formic acid (70%). Upon drying, 1 µL of α-cyano-4-hydroxycinnamic acid was spotted onto the samples. Each isolate was analyzed twice and *E. coli* DH5α was used as a control in each experiment. Spectra were acquired using the MicroflexLT system (Bruker Daltonics) and the resulting mass spectra were compared to the references in the database using the MALDI Biotyper 7.0 (Bruker^®^, 2019) program.

### 2.3. Antimicrobial Susceptibility Testing

Antimicrobial susceptibility of the 53 bacterial isolates was determined for automated systems using the BD PHOENIX (Enzipharma Diagnóstica, Rio de Janeiro, Brazil) and VITEK2 (BioMérieux, Marcy-l′Étoile, France) equipment, following the manufacturers’ instructions and by the Disk-Diffusion method according to American guideline [8] and the Brazilian BrCAST manual [9]. Suspensions were obtained from colonies in a saline solution (NaCl) at 0.85% (*m*/*v*), adjusting the turbidity to the Kirby-Bauer standard, equivalent to 0.5 on the McFarland scale (corresponding to a bacterial concentration of approximately 10^8^ CFU/mL. The selection of the 25 antimicrobials was based on their frequent use in the empirical treatment of UTIs and their inclusion in the CLSI and BrCAST guidelines for standardized susceptibility testing. The following agents were included in the disks used for testing: Nalidixic Acid (NAL 30 μg), Amikacin (AMI 30 μg), Amoxicillin + Clavulanic Acid (AMC 30 μg), Ampicillin (AMP 10 μg), Ampicillin/Sulbactam (APS 20 μg), Aztreonam (ATM 30 μg), Cefazolin (CFZ 30 μg), Cefepime (CPM 30 μg), Cefotaxime (CTX 30 μg), Cefoxitin (CFO 30 μg), Ceftriaxone (CRO 30 μg), Cefuroxime (CRX 30 μg), Ciprofloxacin (CIP 5 μg), Ertapenem (ERT 10 μg), Fosfomycin (FOS 200 μg), Gentamicin (GEN 10 μg), Imipenem (IPM 10 μg), Levofloxacin (LVX 5 μg), Meropenem (MPM 10 μg), Nitrofurantoin (NIT 300 μg), Norfloxacin (NOR 10 μg), Piperacillin/Tazobactam (PIT 110 μg), Trimethoprim/Sulfamethoxazole (SUT 25 μg), Tetracycline (TET 30 μg), and Tobramycin (TOB 10 μg). To compare the obtained results, Cohen’s Kappa coefficient and the percentage agreement of the results were calculated using the Jamovi^®^ software (version 2.5) retrieved from https://www.jamovi.org (accessed on 10 December 2024). The Kappa coefficient was used to measure the agreement between two or more ratings qualitatively, accounting for chance. A Kappa value close to 1 indicates almost perfect agreement, while a value close to 0 suggests random agreement [10]. Kappa values were interpreted as follows: <0.2 (poor agreement), 0.21–0.40 (fair), 0.41–0.60 (moderate), 0.61–0.80 (substantial), and >0.81 (almost perfect). The chi-squared test was also used to evaluate statistically significant differences, considering a confidence interval of 95%.

## 3. Results and Discussion

Disk diffusion testing is a widely used method for assessing bacterial susceptibility to antimicrobial agents. CLSI, EUCAST, and BrCAST provide guidelines outlining specific recommendations for disk content, including the concentration of antibiotics embedded in test disks. However, divergences between these guidelines can affect the interpretation of inhibition zone sizes, potentially leading to discrepancies in result comparisons across standards [4,11]. Transitioning from CLSI to BrCAST can result in misleading alterations to epidemiological susceptibility profiles. Recent research indicates that using BrCAST may increase the classification of certain isolates as resistant, significantly impacting treatment decisions and public health policies [4].

The present study shows, for the first time, a comparative analysis of the antimicrobial susceptibility determined by disk diffusion test (manual method) and by two automated systems (PHOENIX BD™ and VITEK2) using two interpretation guidelines (CLSI and BrCAST), focusing on resistance and susceptibility rates from isolates causing community-acquired urinary tract infections (Table 1).

All bacterial isolates were identified as *Escherichia coli* by MALDI-TOF MS (53; 100%). When evaluating overall antimicrobial susceptibility, BrCAST reported 100% UPEC isolates as being susceptible to AMI, while CLSI registered 73.58% as susceptible, 5.66% as resistant and classified 20.75% of them in the intermediate category. Analyzing each class or antimicrobial agent individually, for AMC, for example, CLSI classified 62.26% of the bacterial isolates as being susceptible, whereas BrCAST showed a percentage of 50.94%; and the resistance percentage for this drug was significantly higher using BrCAST criteria (49.06%) when compared to CLSI (9.43%). For AMP, both CLSI and BrCAST classified more than 69% of the UPEC isolates as resistant, but CLSI allocated 7.55% of them in the intermediate category, which is absent in the BrCAST, creating a discrepancy (Table 1).

For carbapenems, specifically ertapenem (ERT) and meropenem (MPM), both CLSI and BrCAST guidelines classified 100% of the *E. coli* isolates as susceptible to these antibiotics. This consistent performance highlights the robustness and reliability of carbapenems in the treatment of resistant infections, particularly those caused by uropathogenic *E. coli* [12]. Their efficacy across varying interpretive criteria reaffirms their critical role as a cornerstone in the therapeutic management of UTIs, especially in cases involving multidrug-resistant strains. The high susceptibility rates also emphasize the importance of preserving carbapenems as a last-resort option to mitigate the risk of resistance development in clinical practice [13].

To the cephalosporins, BrCAST indicated a higher resistance rate for CFZ (64.15%) and CRX (64.29%) when compared to CLSI (45.28% and 30.36%, respectively). In contrast, to FOS and NIT, more than 85% of the UPEC isolates presented susceptibility by both guidelines, showing that these antibiotics can be good option of drugs to UTI treatment, particularly when conventional antibiotics face resistance.

The concordance index (C%) between CLSI and BrCAST is crucial for interpreting results. Antibiotics such as ERT and MPM showed 100% concordance, while CFZ exhibited a much lower rate (57%). The high resistance rates for some antimicrobial agents in both methods (e.g., AMP and APS) highlight the importance of practicing empirical antibiotic therapy with caution in UTI treatments and prioritizing microbiological analysis. Conversely, FOS and carbapenems seem to be reliable choices for UTI treating by resistant UPEC [14].

This study analyzed automated methods, which minimize human error during analysis. The PHOENIX BD™ method, based on the CLSI manual, and the VITEK^®^ 2 system, based on the BrCAST manual, were evaluated. Table 2 and Table 3 present the results observed between the automated and manual methods. While the PHOENIX BD offers automation and potentially faster results, the disk diffusion method based on CLSI is widely used and occasionally provides more conservative results regarding susceptibility and resistance [15]. The choice between methods should consider concordance for each specific antimicrobial (Table 2).

For AMI, the PHOENIX method reported higher susceptibility (96.23%) compared to the disk diffusion method (73.58%). Resistance and intermediate responses were recorded solely in the manual method, with a concordance of 70% and a low Kappa (0.325), suggesting poor agreement. For AMP, the PHOENIX system detected more susceptible isolates (45.28%) than the disk diffusion method (22.64%). However, a discrepancy in resistance percentages was noted: 69.81% for the manual method versus 50.94% for PHOENIX, resulting in a concordance of 59% and a Kappa of 0.246, indicating considerable discordance between methods.

For CPM, both methods presented similar susceptibility percentages, with PHOE-NIX (79.25%) slightly exceeding disk diffusion (73.58%). Resistance rates were consistent between methods, showing a concordance of 68% and a Kappa of 0.205. Results for Cefoxitin were closely aligned across both methods, demonstrating high concordance (91%) and a substantial Kappa (0.78), indicating good consistency. CIP also yielded similar results, with a concordance of 68% and a Kappa of 0.409, reflecting moderate agreement.

For ERT and MPM, the disk diffusion method reported 100% susceptibility, while the PHOENIX system detected slightly fewer susceptible isolates (88.68% for ERT and 96.23% for MPM). Both methods exhibited high concordance above 89% and strong Kappa values (greater than 0.8). Concordance was similarly high (84%) for PIT, although the PHOENIX method reported no resistance, whereas disk diffusion indicated 3.77% resistance.

The PHOENIX method reported more susceptible isolates (62.26%) compared to disk diffusion (54.72%), while resistance was higher in the manual method for SUT, yielding reasonable concordance (60%) with a Kappa of 0.672. For antibiotics like Cefoxitin, ERT, IPM, and PIT, both methods demonstrated good concordance, suggesting reliability in susceptibility determination. However, greater discordance was evident for agents such as AMI and AMP, reflected in low Kappa values, indicating potential discrepancies in results. The PHOENIX system tended to report higher susceptibility and lower resistance for several antimicrobials compared to CLSI disk diffusion, which may be relevant for guiding clinical decisions [15].

Table 3 summarizes the antimicrobial susceptibility data obtained through the manual method compared to the automated VITEK2 method across various antibiotics. Notably, both methods reported 100% susceptibility for AMI, indicating its robust efficacy against the tested strains. In the case of AMC, BrCAST demonstrated a susceptibility of 50.94% and resistance of 49.06%, while VITEK2 showed slightly lower susceptibility at 50.94% with an equal resistance rate. This notable discrepancy in resistance rates emphasizes the potential impact on clinical decision-making. Similarly, CPM and CRO presented comparable results: both methods reported 73.58% susceptibility and 26.42% resistance for CPM. However, a reduction in CRO susceptibility to 69.81% was noted with VITEK2, highlighting variations in susceptibility interpretations between the two methodologies.

The evaluation of CRX revealed a consensus on its high resistance, with both methods reporting 0% susceptibility along with 35.85% resistance and 64.15% of isolates classified as intermediate. This agreement underscores the prevailing resistance associated with CRX. For CIP, BrCAST reported 58.49% susceptibility against 37.74% resistance, while VITEK2 recorded 54.72% susceptibility with the same resistance rate, suggesting consistency in the resistance profiles. Importantly, both methods confirmed 100% susceptibility for ERT and MPM, reinforcing the continued efficacy of carbapenems against the strains assessed in this study.

Further analysis of NIT indicated 88.68% susceptibility with BrCAST, contrasting with VITEK2’s 100%, which may suggest variability in efficacy. For GEN, BrCAST re-ported 75.47% susceptibility compared to VITEK2’s higher 88.68%, highlighting a significant difference in resistance rates. Lastly, PIT demonstrated 88.68% susceptibility via BrCAST, while VITEK2 indicated a slight decrease to 86.79%. In evaluating SUT, BrCAST revealed a susceptibility of 54.72% with a high resistance rate of 45.28%, contrasting with VITEK2’s lower susceptibility of 43.40%. These findings illustrate the critical need for consistent antimicrobial susceptibility testing methodologies to guide effective treatment decisions [16].

These results highlight the importance of considering the differences in susceptibility and resistance rates between the methods, which may have significant implications for treatment choice and infection management. Overall, the concordance between the two methods was high, especially for critical antibiotics such as AMI, ERT, and MPM, but the discrepancies observed in other agents, such as AMC and GEN, need further investigation to optimize treatment strategies.

CLSI and BrCAST offer guidelines for antimicrobial susceptibility testing, but they differ in some key aspects (Table 4). Understanding these differences is crucial for laboratories and clinicians to ensure accurate interpretation of susceptibility profiles. The specific values used to categorize isolates as susceptible or resistant differ between the two methods, with BrCAST often employing more stringent breakpoints, resulting in higher rates of resistance classification compared to CLSI. This can lead to significant variations in susceptibility profiles for specific pathogens [17].

To date, there are only a few publications comparing the CLSI and BrCAST manuals. Notably, no studies have specifically compared the cut-off points provided by BrCAST and CLSI for antimicrobial agents used to treat urinary tract infections (UTIs) caused by uropathogenic *Escherichia coli* (UPEC). However, differences in susceptibility profiles have been observed for common pathogens such as *Pseudomonas aeruginosa* and *Staphylococcus aureus*, with BrCAST often reporting higher susceptibility rates to certain antibiotics [4,11].

A study on Coagulase-negative staphylococci (CoNS) isolates, published in 2020, highlighted changes in the interpretation of antimicrobial susceptibility tests following the implementation of BrCAST/EUCAST guidelines in 2019. These changes introduced new national standards and affected the resistance profiles of CoNS isolates [4]. The CoNS study employed a methodology similar to ours, and its findings on resistance align with our results for UPEC, where the prevalence of multidrug-resistant isolates increased under BrCAST guidelines.

As we have discussed here, the authors of the CoNS study similarly concluded that adopting BrCAST/EUCAST guidelines inevitably leads to shifts in epidemiological susceptibility profiles. They emphasized the importance of clinicians, institutions, and clinical and research microbiology laboratories being aware of the potential implications of these changes [4,8,9,10,11].

In this study, we prioritized phenotypic methods commonly used in clinical practice, such as the disk diffusion method and automated systems (PHOENIX BD™ and VITEK2), with interpretations based on CLSI and BrCAST guidelines. This approach reflects real-world laboratory settings, especially in resource-limited regions, where advanced molecular tools are often unavailable due to their higher costs and infrastructure requirements. While genetic testing offers unparalleled precision in identifying resistance determinants, it requires significant financial investment and technical expertise, which may not be feasible for many underdeveloped and developing countries [18]. Also, one significant limitation of molecular testing is that the detection of a resistance gene does not necessarily confirm its expression. In other words, the presence of a resistance gene does not guarantee that it is actively contributing to the resistant phenotype in the tested bacteria [6].

Incorporating genetic testing into future studies would significantly enhance our understanding of resistance mechanisms by identifying key genes associated with resistance, such as those encoding extended-spectrum β-lactamases (ESBLs) or carbapenemases. Combining genotypic and phenotypic testing provides a complementary approach, offering a more comprehensive and accurate characterization of antimicrobial resistance. However, it is important to note that the widespread adoption of such testing remains challenging in many regions due to economic and logistical barriers [18,19]. The current study contributes to the field of clinical microbiology and antimicrobial resistance monitoring by focusing on phenotypic methods that are more cost-effective and widely applicable in clinical settings, particularly in low-resource environments. Our findings emphasize the critical impact of interpretive guidelines (CLSI vs. BrCAST) on susceptibility classifications and clinical decision-making, highlighting the need for standardized, accessible testing methods to optimize treatment strategies.

## 4. Conclusions

This study highlights several discrepancies in the results observed according to the CLSI and BrCAST guidelines in determining the antimicrobial susceptibility of UPEC isolates to 13 antimicrobial drugs (AMC, APS, CFZ, CPM, CFO, CRX, FOS, GEN, LVX, NOR, PIT, TET, and TOB), which may influence decisions in UTI treatment and clinical outcomes.

Carbapenems such as ERT and MPM were consistently effective across both methods, underscoring their reliability in treating UTI by UPEC resistant isolates. Additionally, the comparison between disk diffusion and automated methods (PHOENIX BD™ and VITEK2) also revealed a great variability in the results. These findings emphasize the importance of selecting appropriate testing methodologies to determine the antimicrobial susceptibility, as well as considering regional guidelines.

Overall, this study underscores the need for continuous evaluation of antimicrobial susceptibility testing practices to optimize UTI treatment strategies and contribute to slowing the advance of antimicrobial resistance and the dissemination of resistant bacteria causing human infections.

## Figures and Tables

**Table 1:** Comparison of susceptibility determination results of Uropathogenic *Escherichia coli* Isolates to antimicrobial agents by Disk diffusion: CLSI vs. BrCAST guidelines.

Antimicrobial Tested	DD	S	S%	R	R%	I	I%	C%	K	*p* Value
Aminoglycosides										
Amikacin (30 μg)	CLSI	39	73.59%	3	5.66%	11	20.75%	73%	0.807	<0.0001
	BrCAST	53	100.00%	0	0.00%	0	0.00%			
Gentamicin (10 μg)	CLSI	42	79.25%	11	20.75%	0	0.00%	95%	0.846	0.6425
	BrCAST	40	75.47%	13	24.53%	0	0.00%			
Tobramycin (10 μg)	CLSI	38	71.70%	14	26.42%	1	1.89%	93%	0.837	0.8406
	BrCAST	36	67.92%	17	32.08%	0	0.00%			
Penicillins										
Ampicilin (10 μg)	CLSI	12	22.64%	37	69.81%	4	7.55%	93%	0.844	0.1206
	BrCAST	16	30.19%	37	69.81%	0	0.00%			
Amoxicillin/Clavulanate (30 μg)	CLSI	33	62.26%	5	9.43%	15	28.30%	63%	0.889	0.2287
	BrCAST	27	50.94%	26	49.06%	0	0.00%			
Ampicillin/Sulbactam (20 μg)	CLSI	23	43.40%	20	37.74%	10	18.87%	82%	0.699	0.1307
	BrCAST	23	43.40%	30	56.60%	0	0.00%			
Piperacillin/Tazobactam (110 μg)	CLSI	44	83.02%	2	3.77%	7	13.21%	88%	0.894	0.0745
	BrCAST	47	88.68%	6	11.32%	0	0.00%			
Cephalosporins										
Cefazoline (30 μg)	CLSI	12	22.64%	24	45.28%	17	32.08%	57%	0.894	0.0319
	BrCAST	0	0.00%	34	64.15%	19	35.85%			
Cefoxitin (30 μg)	CLSI	50	94.34%	1	1.89%	2	3.77%	82%	0.843	0.1525
	BrCAST	42	79.25%	11	20.75%	0	0.00%			
Cefuroxime (30 μg)	CLSI	37	69.81%	14	26.42%	2	3.77%	27%	0.169	<0.0001
	BrCAST	0	0.00%	19	35.85%	34	64.15%			
Cefotaxime (30 μg)	CLSI	38	71.70%	15	28.30%	0	0.00%	96%	0.915	0.8380
	BrCAST	38	71.70%	14	26.42%	1	1.89%			
Ceftriaxone (30 μg)	CLSI	39	73.58%	14	26.42%	0	0.00%	100%	1	>0.9999
	BrCAST	39	73.58%	14	26.42%	0	0.00%			
Cefepime (30 μg)	CLSI	39	73.58%	8	15.09%	6	11.32%	88%	0.706	0.3111
	BrCAST	39	73.58%	14	26.42%	0	0.00%			
Carbapenems										
Ertapenem (10 μg)	CLSI	53	100.00%	0	0.00%	0	0.00%	100%	1	>0.9999
	BrCAST	53	100.00%	0	0.00%	0	0.00%			
Imipenem (10 μg)	CLSI	52	98.11%	0	0.00%	1	1.89%	2%	0	<0.0001
	BrCAST	0	0.00%	0	0.00%	53	100.00%			
Meropenem (10 μg)	CLSI	53	100.00%	0	0.00%	0	0.00%	100%	1	>0.9999
	BrCAST	53	100.00%	0	0.00%	0	0.00%			
Monobactams										
Aztreonam (30 μg)	CLSI	38	71.70%	12	22.64%	3	5.66%	91%	0.8	0.8640
	BrCAST	36	67.92%	15	28.30%	2	3.77%			
Fluoroquinolones										
Ciprofloxacin (5 μg)	CLSI	29	54.72%	20	37.74%	4	7.55%	96%	0.933	0.5191
	BrCAST	31	58.49%	20	37.74%	2	3.77%			
Levofloxacin (5 μg)	CLSI	32	60.38%	18	33.96%	3	5.66%	98%	0.965	0.8679
	BrCAST	32	60.38%	19	35.85%	2	3.77%			
Norfloxacin (10 μg)	CLSI	35	66.04%	17	32.08%	1	1.89%	88%	0.749	0.3409
	BrCAST	29	54.72%	24	45.28%	0	0.00%			
Nitrofurans										
Nitrofurantoin (300 μg)	CLSI	47	88.68%	6	11.32%	0	0.00%	100%	1	>0.9999
	BrCAST	47	88.68%	6	11.32%	0	0.00%			
Phosphonic acids										
Phosphomicin (200 μg)	CLSI	47	88.68%	6	11.32%	0	0.00%	96%	0.837	0.7672
	BrCAST	46	86.79%	7	13.21%	0	0.00%			
Sulfonamides										
Sulfazotrim (25 μg)	CLSI	29	54.72%	24	45.28%	0	0.00%	100%	1	>0.9999
	BrCAST	29	54.72%	24	45.28%	0	0.00%			
Tetracyclines										
Tetracycline (30 μg)	CLSI	24	45.28%	28	52.83%	1	1.89%	98%	0.965	0.8505
	BrCAST	24	45.28%	29	54.72%	0	0.00%			

DD: Disk Diffusion; S: Number of isolates susceptible to the tested antimicrobial; S%: Percentage of susceptible isolates; R: Number of resistant isolates; R%: Percentage of resistant isolates; I: Number of isolates with intermediate susceptibility; I%: Per-centage of isolates with intermediate susceptibility; C%: Concordance between CLSI and BrCAST guidelines for each tested anti-microbial; K: Kappa value, indicating the degree of agreement between the two testing methods. Kappa values were interpreted as follows: <0.2 (poor agreement), 0.21–0.40 (fair), 0.41–0.60 (moderate), 0.61–0.80 (substantial), and >0.81 (almost perfect). All bacterial isolates were identified as *Escherichia coli* by MALDI-TOF MS (53; 100%).

**Table 2:** Comparative analysis of antimicrobial susceptibility results: manual DD/CLSI (Disk Diffusion) vs. automated PHOENIX BD methods.

Antimicrobial Tested	DD	S	S%	R	R%	I	I%	C%	K	*p* Value
Aminoglycosides										
Amikacin (30 μg)	DD/CLSI	39	73.58%	3	5.66%	11	20.75%	70	0.325	0.0022
	PHOENIX	51	96.23%	0	0.00%	2	3.77%			
Gentamicin (10 μg)	DD/CLSI	42	79.25%	11	20.75%	0	0.00%	71	0.705	0.6866
	PHOENIX	43	81.13%	7	13.21%	3	5.66%			
Penicillins										
Ampicilin (10 μg)	DD/CLSI	12	22.64%	37	69.81%	4	7.55%	59	0.246	0.0156
	PHOENIX	24	45.28%	27	50.94%	2	3.77%			
Piperacillin/Tazobactam (110 μg)	DD/CLSI	44	83.02%	2	3.77%	7	13.21%	84	0.862	0.0403
	PHOENIX	51	96.23%	0	0.00%	2	3.77%			
Cephalosporins										
Cefoxitin (30 μg)	DD/CLSI	50	94.34%	1	1.89%	2	3.77%	91	0.780	0.5352
	PHOENIX	49	92.45%	0	0.00%	4	7.55%			
Cefepime (30 μg)	DD/CLSI	39	73.58%	8	15.09%	6	11.32%	68	0.205	0.4481
	PHOENIX	42	79.25%	7	13.21%	4	7.55%			
Carbapenems										
Ertapenem (10 μg)	DD/CLSI	53	100.00%	0	0.00%	0	0.00%	89	0.801	0.0117
	PHOENIX	47	88.68%	0	0.00%	6	11.32%			
Imipenem (10 μg)	DD/CLSI	52	98.11%	0	0.00%	1	1.89%	95	0.789	0.5581
	PHOENIX	51	96.23%	0	0.00%	2	3.77%			
Meropenem (10 μg)	DD/CLSI	53	100.00%	0	0.00%	0	0.00%	96	0.856	0.1534
	PHOENIX	51	96.23%	0	0.00%	2	3.77%			
Fluoroquinolones										
Ciprofloxacin (5 μg)	DD/CLSI	29	54.72%	20	37.74%	4	7.55%	68	0.409	0.7533
	PHOENIX	30	56.60%	20	37.74%	3	5.66%			
Levofloxacin (5 μg)	DD/CLSI	32	60.38%	18	33.96%	3	5.66%	100	1	>0.9999
	PHOENIX	32	60.38%	18	33.96%	3	5.66%			
Sulfonamides										
Sulfazotrim (25 μg)	DD/CLSI	29	54.72%	24	45.28%	0	0.00%	60	0.672	0.7152
	PHOENIX	33	62.26%	18	33.96%	2	3.77%			

DD: Disk Diffusion; S: Number of isolates susceptible to the tested antimicrobial; S%: Percentage of susceptible isolates; R: Number of resistant isolates; R%: Percentage of resistant isolates; I: Number of isolates with intermediate susceptibility; I%: Per-centage of isolates with intermediate susceptibility; C%: Concordance between DD/CLSI and PHOENIX methods for each tested antimicrobial; K: Kappa value, indicating the degree of agreement between the two testing methods. Kappa values were interpreted as follows: <0.2 (poor agreement), 0.21–0.40 (fair), 0.41–0.60 (moderate), 0.61–0.80 (substantial), and >0.81 (almost perfect). All bacterial isolates were identified as *Escherichia coli* by MALDI-TOF MS (53; 100%).

**Table 3:** Comparison of antimicrobial susceptibility data between the manual TSA/BrCAST method and the automated VITEK2 method for various antibiotics.

Antimicrobial Tested	DD	S	S%	R	R%	I	I%	C%	K	*p* Value
Aminoglycosides										
Amikacin (30 μg)	DD/BrCAST	53	100.00%	0	0.00%	0	0.00%	100	1	>0.9999
	VITEK2	53	100.00%	0	0.00%	0	0.00%			
Gentamicin (10 μg)	DD/BrCAST	40	75.47%	13	24.53%	0	0.00%	81	0.878	0.0763
	VITEK2	47	88.68%	6	11.32%	0	0.00%			
Penicillins										
Amoxicillin/Clavulanate (30 μg)	DD/BrCAST	27	50.94%	26	49.06%	0	0.00%	100	1	>0.9999
	VITEK2	27	50.94%	26	49.06%	0	0.00%			
Piperacillin/Tazobactam (110 μg)	DD/BrCAST	47	88.68%	6	11.32%	0	0.00%	89	0.812	0.7672
	VITEK2	46	86.79%	7	13.21%	0	0.00%			
Cephalosporins										
Cefuroxime (30 μg)	DD/BRCAST	0	0.00%	19	35.85%	34	64.15%	100	1	0.8385
	VITEK2	0	0.00%	18	33.96%	35	66.04%			
Ceftriaxone (30 μg)	DD/BrCAST	39	73.58%	14	26.42%	0	0.00%	87	0.845	0.5397
	VITEK2	37	69.81%	15	28.30%	1	1.89%			
Cefepime (30 μg)	DD/BrCAST	39	73.58%	14	26.42%	0	0.00%	85	0.806	0.4818
	VITEK2	40	75.47%	8	15.09%	5	9.43%			
Carbapenems										
Ertapenem (10 μg)	DD/BrCAST	53	100.00%	0	0.00%	0	0.00%	100	1	>0.9999
	VITEK2	53	100.00%	0	0.00%	0	0.00%			
Meropenem (10 μg)	DD/BrCAST	53	100.00%	0	0.00%	0	0.00%	100	1	>0.9999
	VITEK2	53	100.00%	0	0.00%	0	0.00%			
Fluoroquinolones										
Ciprofloxacin (5 μg)	DD/BrCAST	31	58.49%	20	37.74%	2	3.77%	81	0.868	0.5191
	VITEK2	29	54.72%	20	37.74%	4	7.55%			
Norfloxacin (10 μg)	DD/BrCAST	29	54.72%	24	45.28%	0	0.00%	86	0.816	0.845
	VITEK2	30	56.60%	23	43.40%	0	0.00%			
Nitrofurans										
Nitrofurantoin (300 μg)	DD/BrCAST	47	88.68%	6	11.32%	0	0.00%	89	0.8	0.0117
	VITEK2	53	100.00%	0	0.00%	0	0.00%			
Sulfonamides										
Sulfazotrim (25 μg)	DD/BrCAST	29	54.72%	24	45.28%	0	0.00%	88	0.864	0.2437
	VITEK2	23	43.40%	30	56.60%	0	0.00%			

DD: Disk Diffusion; S: Number of isolates susceptible to the tested antimicrobial; S%: Percentage of susceptible isolates; R: Number of resistant isolates; R%: Percentage of resistant isolates; I: Number of isolates with intermediate susceptibility; I%: Percentage of isolates with intermediate susceptibility; C%: Concordance between DD/BrCAST and VITEK2 methods for each tested antimicrobial; K: Kappa value, indicating the degree of agreement between the two testing methods. Kappa values were interpreted as follows: <0.2 (poor agreement), 0.21–0.40 (fair), 0.41–0.60 (moderate), 0.61–0.80 (substantial), and >0.81 (almost perfect). All bacterial isolates were identified as *Escherichia coli* by MALDI-TOF MS (53; 100%).

**Table 4:** Summary of key differences between CLSI and BrCAST criteria for interpreting antimicrobial susceptibility testing results.

Feature	CLSI	BrCAST
Accessibility	Requires annual subscription fees (approx. $500 for non-members) [8,17].	Freely available as part of the EUCAST guidelines [9,17].
Methodology	Based on Minimum Inhibitory Concentrations (MICs), pharmacokinetics, and resistance mechanisms [8,17].	Similar approach to EUCAST, focusing on epidemiological cut-offs (ECOFFs) [4,9].
Implementation Date	Established guidelines have been in place for many years, with updates [4,8].	Adopted as the national standard in Brazil in 2019 [4,9].
Impact on Susceptibility Profiles	Variability in categorization; may lead to underestimation of resistance in certain pathogens [4,8].	Increased categorization of intermediate susceptibility (I) and resistant results [4,9].
Specific Pathogen Findings	Significant differences noted in susceptibility rates for drugs like cefepime and imipenem compared to BrCAST [4,8].	Higher rates of multidrug-resistant isolates reported under BrCAST guidelines, particularly for coagulase-negative staphylococci [4,9].
Decision-Making Process	Involves industry representatives in decision-making; less transparency [8,17].	Industry has a consultative role only; promotes inclusivity through National Antimicrobial Susceptibility Testing Committees [9,17].

## Data Availability

The original contributions presented in this study are included in the article. Further inquiries can be directed to the corresponding authors.
